# What about the “Self” is Processed in the Posterior Cingulate Cortex?

**DOI:** 10.3389/fnhum.2013.00647

**Published:** 2013-10-02

**Authors:** Judson A. Brewer, Kathleen A. Garrison, Susan Whitfield-Gabrieli

**Affiliations:** ^1^Department of Psychiatry, Yale University, New Haven, CT, USA; ^2^Department of Brain and Cognitive Sciences, Massachusetts Institute of Technology, Cambridge, MA, USA

**Keywords:** default mode network, real-time fMRI, meditation, posterior cingulate cortex, self-referential processing, mind wandering, resting state, craving

## Abstract

In the past decade, neuroimaging research has begun to identify key brain regions involved in self-referential processing, most consistently midline structures such as the posterior cingulate cortex (PCC). The majority of studies have employed cognitive tasks such as judgment about trait adjectives or mind wandering, that have been associated with increased PCC activity. Conversely, tasks that share an element of present-centered attention (being “on task”), ranging from working memory to meditation, have been associated with decreased PCC activity. Given the complexity of cognitive processes that likely contribute to these tasks, the specific contribution of the PCC to self-related processes still remains unknown. Building on this prior literature, recent studies have employed sampling methods that more precisely link subjective experience to brain activity, such as real-time fMRI neurofeedback. This recent work suggests that PCC activity may represent a sub-component cognitive process of self-reference – “getting caught up in” one’s experience. For example, getting caught up in a drug craving or a particular viewpoint. In this paper, we will review evidence across a number of different domains of cognitive neuroscience that converges in activation and deactivation of the PCC including recent neurophenomenological studies of PCC activity using real-time fMRI neurofeedback.

## Introduction

Over a decade ago, using the simple task instruction of “lie still and don’t do anything in particular,” Raichle et al. (2001) discovered that the posterior cingulate cortex (PCC) was functionally coupled with other brain regions now considered the default mode network (DMN). Numerous studies have since implicated the PCC in a host of functions ranging from those that elicit activation such as mind wandering, social cognition, and drug craving, to those that elicit deactivation such as focused attention and meditation. Many of these studies have interpreted the findings in terms of the PCC being involved in self-related aspects of cognitive processing. However, it is still unclear what aspects of the “self” are processed in the PCC. Given the growing body of evidence on PCC function from different domains, including self-related processing, social cognition, and addiction, among others, it may now be possible to identify potential phenomenological descriptors that are common across domains. In this paper, we propose that PCC activity may be related to a cognitive process of being “attached to” or “caught up” in one’s experience. We will describe being “caught up” in experience, and then discuss evidence from cognitive and clinical neuroscience that provides a basis for this hypothesis. We will first discuss findings that PCC activation is related to being “caught up in” experience, including self-related and social cognitive processing, disruption of attention, and craving. We will then discuss findings that PCC deactivation is related to not being “caught up in” experience, including present-centered awareness or attention. For the purpose of this review, we will focus on a specific functionally defined sub-region of the PCC most associated with the DMN (Leech et al., 2012), though this brain region likely supports other cognitive functions as well. Additionally, we will focus our discussion on studies measuring activity rather than functional connectivity; though the latter is related in an important way, it is beyond the scope of this paper and has been recently reviewed elsewhere (e.g., Whitfield-Gabrieli and Ford, 2012). Finally, we will explore PCC activity as a possible marker of getting “caught up in” experience, pointing to a likely larger network of brain regions involved in this cognitive process.

### What does it mean to be “caught up in” experience?

We have all been caught up in experience – whether positive or negative. This can happen when we have a disagreement with a loved one or colleague that goes on and on to the point where we don’t even remember what we were arguing about – we get attached to a certain viewpoint, or even just “being right” and can’t let go no matter how ridiculous the argument becomes. We can also get caught up in something by being pulled into our experience; for example, we start an internet search for something, get distracted by something else that looks interesting, then something else, and on and on until we find that we are on some random website and don’t remember how we got there. Though there are likely differences between getting caught up in positive or negative experiences, there may be a shared experiential component; we will include both of these in the broader category of getting caught up in experience as a first pass at identifying neural correlates therein. In fact, there is precedent for brain regions subserving oppositely valenced affective experience, as has been seen in previous studies of appetitive and aversive stimuli (Carlezon and Thomas, 2009).

Being caught up in experience can be noticeable; for instance we notice that we contract when someone is yelling at us. At other times, the experience of being caught up may be subtle, or we may be so engrossed – as is with the case of daydreaming – that we aren’t aware that we are caught up until after the experience has passed. Though being caught up in experience may be common from an experiential standpoint, from a neuroscientific framework, it likely involves a number of overlapping cognitive processes, including self-referential/internally oriented networks, emotion processing, social cognition, and evaluative/judgment systems among others. As each of these systems in turn involves complex networks of brain regions, it may be helpful to look across multiple cognitive domains to identify a common experiential element. Is the PCC a good candidate brain region to begin this exploration? In the following sections, we will give brief experiential examples of being caught up in one’s experience, and explore related cognitive domains and their convergence in neural activation patterns.

## PCC Activation is Related to Being Caught Up in Mental Content

### PCC activity is associated with self-related processing

#### What is it like if someone asks if you think you’re “outgoing,” “patient,” or “nosy”? Are we attached to certain concepts of ourselves? Do we get caught up in these evaluations? What is this experience like and how does this map onto our brain activity?

Aside from studies of the resting state, perhaps the best-studied category of cognitive tasks that activate the PCC are those involving self-related processing. Early work by Kelley et al. (2002) used a simple task of presenting trait adjectives to subjects during fMRI and asking “Does the adjective describe you?” (“self” condition), or for comparison, “Does the adjective describe current U.S. President George Bush?” (“other” condition). Relatively greater PCC activity was found for the “self” as compared to the “other” condition (Kelley et al., 2002). These findings have been since replicated (e.g., Heatherton et al., 2006) and extended using other sensory modalities such as aural presentation of adjectives (Johnson et al., 2002) or reflective self-awareness of personality and physical appearance (Kjaer et al., 2002). A meta-analysis performed by Northoff et al. (2006) concluded that midline structures including the PCC and medial prefrontal cortex (mPFC) comprise a “core,” “mental,” or “minimal” self (Northoff et al., 2006).

In this meta-analysis (Northoff et al., 2006), Northoff also speculated that overlap between self-referential and resting state processing should include predominantly interoceptive stimuli. This assertion has gained empirical support in recent years. For example, studies using experience sampling have found that close to 50% of waking life is spent mind wandering to past and future events (Killingsworth and Gilbert, 2010). Mind wandering has been shown to activate the PCC (Weissman et al., 2006; Mason et al., 2007) as do cognitive tasks that elicit future oriented thinking (Andrews-Hanna et al., 2010). These findings suggest that there is an experiential default mode (mind wandering) associated with PCC activity. Bringing these findings together, Whitfield-Gabrieli et al. (2011) directly compared task-independent resting state with task-dependent self-related processing during evaluation of trait adjectives and found a distinct convergence of brain activations in the PCC and mPFC.

Most studies of self-related processing find activations in both the PCC and mPFC, brain regions that have also been shown to be tightly functionally coupled (Fox et al., 2005; Andrews-Hanna et al., 2010). Anatomical and functional studies have begun to distinguish the roles of the PCC and mPFC in self-related processing. For example, the mPFC appears to integrating information gathered from the internal and external environment and relay it to the PCC (Ongur and Price, 2000; Ongur et al., 2003). Functional imaging studies of the classic hallucinogen psilocybin have found that psilocybin leads to decreased functional coupling of the PCC and mPFC and increased coupling between the mPFC and task-positive brain regions such as the dorsolateral prefrontal cortex (dlPFC) (Carhart-Harris et al., 2012). Psilocybin ingestion is reported to induce an “egoless” or “selfless” state where the boundary between self and other is blurred. One interpretation of these findings is that decreased coupling between the PCC and mPFC with psilocybin corresponds with the subjective experience of a less egoic state, or less “self.”

However, recent meta-analyses by Legrand and Ruby (2009) and Qin and Northoff (2011) have suggested that a more subtle process than just the subjective experience of “self” may be occurring in the PCC. Legrand and Ruby suggested that familiarity with an object might drive PCC activation rather than self-reference. Qin directly tested this by comparing results from imaging studies of self, familiarity, other, and rest. Interestingly, all four categories showed PCC activation! When contrasted, the self category showed robust mPFC activation relative to familiarity, other, and rest, suggesting that there may be a specific cognitive aspect of self-referential processing that is mediated by the mPFC. So what do these four task categories have in common? Agreeing with Legrand and Ruby, Qin suggested that regions such as the PCC may serve as a general evaluation or judgment system.

Consistent with this interpretation, additional self-related processing tasks that may tap into the construct of evaluation or judgment have been found to elicit PCC activity. For example, Johnson et al. (2006) compared reflection on promotion goals (make good things happen) and prevention goals (keep bad things from happening) and found that the PCC was activated by both conditions, but more so by prevention goals. In a related study, Strauman et al. (2012) found that prevention goal priming was specifically associated with PCC activity irrespective of the degree of negative valence of the prompt. If not negative valence, what is it about prevention goals that preferentially activates the PCC? Johnson speculated that PCC activity may be more related to differences in social significance, representational context, or aspects of subjective experience of self, among others. Another study used a self-evaluation task in which individuals chose between two music CDs that they had previously rated as equivalent, and then reported on how much they liked the chosen CD (a phenomenon termed “choice justification”). PCC activity was associated with an increase in liking for chosen CDs, but not a decrease in liking for rejected CDs (Salmoirago-Blotcher et al., 2013). Again, the valence of self-related processing did not specifically relate to PCC activity.

Together these studies support Legrand and Ruby and Qin’s suggestions that evaluation or judgment of experience may be represented in PCC activation – “ought to” goals are often more evaluative than promotion goals, and this may be similar to choice justification, where we get caught up in defending our choices, even to ourselves. If evaluation or judgment overlaps with being caught up in experience, it may provide a parsimonious explanation for how these findings line up with the decreased functional coupling found with psilocybin – the mPFC may subserve more cognitive elements of self, while the PCC functions to evaluate or judge how one *relates* to one’s experience: how much they are caught up in it. If this is the case, this relational aspect should find overlap with other domains of experience.

### PCC activity is associated with social cognitive processing

#### What is it like when we see a co-worker take credit for another’s work? What is it like when someone asks us for spare change on the street? Is there a common social cognitive process whereby we get caught up in moral dilemmas?

Recent work in cognitive neuroscience has demonstrated a role for the PCC in social processing, such as mentalizing, evaluative judgments, and sensitivity to moral issues, among others. A recent meta-analysis of neuroimaging studies in social cognition (Sperduti et al., 2012) found consistent PCC activation related to internal and external agency attribution, perspective taking, observing social interactions, self-related thinking, and causal attribution of social events. For example, a study by van Veen et al. (2009) used an induced compliance procedure in which subjects made a series of false statements to mislead an innocent person to generate cognitive dissonance, and found activity in the dorsal anterior cingulate, anterior insula, and PCC, possibly representing cognitive conflict, negative emotional arousal, and self-related processing, respectively. In another fMRI study (Arsenault et al., 2013), PCC activity correlated with attributional evaluation processing of valenced sentences describing socially relevant everyday situations, more so in the right PCC for positive sentences, and more so in the left PCC for negative sentences. These findings suggest that PCC activity is related to social evaluation.

Another aspect of social cognitive processing shown to engage the PCC is moral dilemma, which may be distinguished as issues related to care, such as benevolence, compassion, and the desire to liberate others from need, or related to justice, such as fairness, impartiality, and the desire to liberate others from injustice. Caceda et al. (2011) presented story segments designed to evoke moral dilemma and found partial neural segregation between care, justice, and neutral issues. The PCC, among other regions, was implicated in processing of both care and justice issues relative to neutral issues. The authors suggested that the purported role of the PCC in autobiographical memory is that interpretive awareness of care issues in moral conflict may be informed by memories of past moral situations, decisions, and outcomes, as well as self-awareness, personal beliefs, and positive emotions. Relatedly, for justice issues, the PCC may modulate predictive social perceptions. Morey et al. (2012) studied guilt and social consequence, and found that the PCC, among other regions, was more strongly activated for actions leading to harm to others relative to oneself, and suggest that actions involving guilt may lead to greater preoccupation with self-actions rather than thoughts about harm caused to others.

These studies suggest that the PCC plays a role in a range of social situations. What is common between moral dilemmas, justice issues, and guilt, among others? What is the experience like when we are faced with moral issues regarding ourselves or others, or guilt that may come as a consequence of our actions? Perhaps similar to self-related processing, there is an element of mental clenching around or being caught up in the experience. It is interesting to note that this appears to occur even for imagined scenarios, such as those in the fMRI studies discussed here.

### PCC activity is associated with disruption of attention

#### What is it like to be interrupted by a text or email notification on your cell phone or computer?

A general task-related decrease of PCC activity has been reported (e.g., Greicius et al., 2003), and task-related increases in PCC activity have been found during lapses of attention. For example, Eichele et al. (2008) found that PCC activity predicts response errors in flanker tasks, and Esposito et al. (2006) found a signal increase and spatial decrease of the PCC activation with working memory load.

Relatedly, PCC activity has been associated with poorer task performance (Wen et al., 2013). For example, increased PCC activity has been associated with lapses in attention that affect task performance, such as in trials preceding errors in a go/no-go task (Li et al., 2007) or with increased reaction time in a demanding task (Weissman et al., 2006; Hinds et al., 2013). In these studies, PCC activity leading to distraction from task performance may reflect mind wandering (Mason et al., 2007). Weissman et al., found that just prior to lapses in attention, the PCC showed increased activity, possibly indicating a shift from the external world to internal mentation. Using real-time fMRI, Hinds et al., found that presenting stimuli during relatively increased DMN activation resulted in significantly slower reaction time compared to presentation during greater activation in the supplementary motor cortex. Another study by Otten and Rugg (2001) used an incidental learning task to study unsuccessful memory encoding, and found greater activation in the PCC and other regions during subsequently forgotten words.

These studies provide evidence for a correlation between increased PCC activity and poorer task performance. With regard to the actual subjective experience of mind wandering or lapses in attention, it is possible that when attention is pulled away from a task, this may manifest as being caught up in the experience, with associated increases in PCC activity.

### PCC activity is associated with craving

#### What is it like to crave a piece of chocolate?

Craving, perhaps one of the most obvious experiences of being caught up in experience, is described clinically and experimentally in terms of desire, urge, want, and need (Tiffany and Wray, 2012); it has been associated with PCC activity in smoking and drug addiction. For example, Brody et al. (2007) showed that smokers resisting cue-induced craving strongly engage the PCC. Similarly, preferential processing of smoking cessation messages highly tailored to the smoker was associated with PCC activity (Chua et al., 2009), possibly in part because the messages were self-related and/or personally meaningful (which also may be more effective at inducing craving). In a case study, a lesion to the PCC led to a disruption of the individual’s nicotine addiction, reported as an immediate loss of cigarette craving, with no urge to smoke at all (Jarraya et al., 2010). Related to this, a larger lesion study (Naqvi et al., 2007) found that smokers with damage to the insula were more likely to quit smoking, associated with loss of urge to smoke, and another study found that increased connectivity between the PCC and the insula in smokers may be attenuated by anti-smoking medications (Carim-Todd et al., 2013).

The relationship between PCC activity and craving has also been reported in studies of drug addiction. In a study by Garavan et al. (2000), cocaine users and cocaine-naïve individuals watched videos of two men smoking crack cocaine to induce cocaine craving during fMRI, leading to activations in the PCC among other regions in cocaine users but not controls. PCC activation was also found in response to watching an evocative sexual video, but not in response to watching a nature video, suggesting that the normal endogenous drive state or craving response may be seated in the PCC (Garavan et al., 2000).

Related to our introductory example of chocolate craving, Yokum and Stice (2013) found reduced activity in the PCC when individuals were asked to think about the long term costs or benefits of eating or not eating and attempt to suppress food cravings, though in this paradigm, it may be difficult to distinguish contributions of the PCC to being “on task” vs. suppressing cravings. Overall, the PCC appears to be involved in aspects of craving, as shown by functional neuroimaging and lesion studies in a number of contexts including smoking, drug addiction, food, and sex. As craving has been specifically described as being caught up in an experience, it may provide the most direct evidence for how being caught up may activate the PCC. With all of these cognitive domains converging in PCC activation, are there opposing cognitive domains that deactivate the PCC, providing complementary evidence for its role in getting caught up in experience?

### PCC deactivation is related to present-centered awareness or attention

#### What is it like to be mindful of the present moment, to allow thoughts to arise without getting caught up in them?

The previous sections have laid out a number of different cognitive experiences that modulate PCC activity. We now turn to studies that show PCC deactivation related to present-centered awareness or attention. In addition to general task-related PCC deactivation, a role for the PCC in getting caught up in experience is supported by the deactivation of this brain region during tasks specifically designed to “not get caught up” such as focused attention or meditation. For example, McKiernan et al. (2003) found that the magnitude of task-induced PCC deactivation increased with task difficulty. Similarly, Wen et al. (2013) found that mean PCC BOLD signal is negatively correlated with accuracy in a spatial visual attention task. Meditation, operationalized for the fMRI setting, may be considered a form of focused attention toward the present moment and away from mind wandering and self-related thinking. In work from our research group, we have found that three types of meditation practices specifically deactivate the PCC in experienced meditators as compared to novices (Brewer et al., 2011). In this study, meditators also reported less mind wandering during meditation than novices. Based on these earlier findings, we have conducted real-time fMRI neurofeedback studies in which we have found that real-time feedback from the PCC corresponds to the subjective experience of mind wandering (increased PCC activity) and focused attention (decreased PCC activity) in meditators and novices, and that meditators are able to volitionally decrease a feedback graph representing PCC activity (Garrison et al., 2013b). Pagnoni (2012) also reported less mind wandering in meditators, as well as a lower relative incidence of elevated PCC activity and better performance on a visual attention task.

A particular advantage of real-time fMRI neurofeedback over standard offline analysis is that it captures variability within blocks of time that would traditionally be regressed to a mean. As cognitive states fluctuate significantly over the course of a 1–3 min block, these transient changes can now be more precisely linked to brain activity to improve characterization therein. We have begun to test the specific hypothesis that the PCC is involved in getting caught up in mental content, using neurophenomenological studies of real-time feedback from the PCC in experienced meditators. In a recent study (Garrison et al., 2013a) meditators were asked to meditate for short (1 min) real-time fMRI runs with feedback from the PCC and immediately report their experience during the meditation. Meditators performed focused attention on the breath meditation while viewing a dynamic feedback graph representing percent signal change in the PCC relative to a baseline task, and were asked to describe their experience during the meditation after each run. Feedback graphs paired with self-reports were analyzed using grounded theory to derive specific testable hypotheses about how PCC activity corresponds to the subjective experience of meditation. Overall, we found that the subjective experience of “undistracted awareness” and “effortless doing” corresponded with PCC deactivation, and “distracted awareness” and “controlling” corresponded with PCC activation. Specifically related to the current review, in many cases meditators reported instances of mind wandering that did not lead to PCC activity, suggesting that the PCC may be involved in something more than the thoughts themselves, such as getting caught up in experience, as suggested by the studies described above.

**Figure 1:**
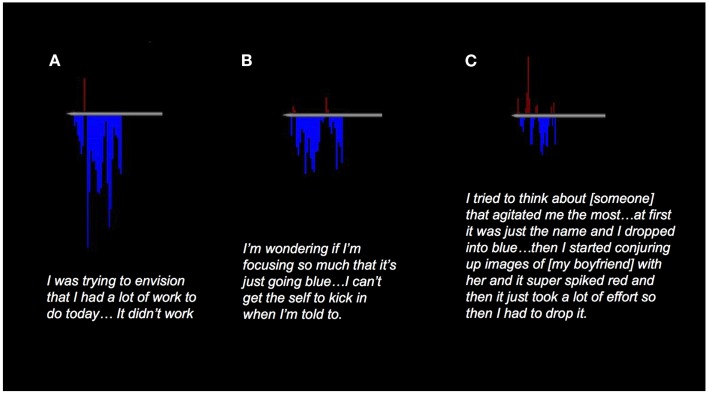
**Examples of real-time neurofeedback from the PCC in meditators**. Graphs show percent signal change in the PCC relative to an active baseline task. **(A)** “I was trying to envision that I had a lot of work to do today … It didn’t work.” **(B)** “I decided to picture wedding plans and so I started off thinking about my wedding and how I wanted to look good and then it just started to go blue. I switched to babies and I thought, ‘I want babies’ and I think that might correlate with a little red blip but then I couldn’t sustain it … I’m wondering if I’m focusing so much that it’s just going blue because I’m focusing but I can’t get, I can’t get the self to kick in when I’m told to.” **(C)** “I tried to think about what was the thing that agitated me most and I thought it was [a certain person] and so I started thinking about her and I, at first it was just the name and I dropped into blue and so and then I started conjuring up images of [my boyfriend] with her and it super spiked and then it just took a lot of effort so then I had to drop it. And I just kept trying to pick it up a little bit which I think correlates with the kind of like final two spikes, the kind of final two points in the red. Although, it was just so much energy, I couldn’t sustain it, which was why I couldn’t keep that really high spike going … I couldn’t sustain it and so that kind of correlates with not being able to hold on to that throughout.”

For example, during a real-time feedback run in which a meditator was asked to increase a feedback graph representing PCC activity, the meditator described being unable to elicit PCC activity by mind wandering: “I was trying to envision that I had a lot of work to do today … It didn’t work” (Figure 1A). Another meditator when trying to activate her PCC reported: “I decided to picture wedding plans and so I started off thinking about my wedding and how I wanted to look good and then it just started to go blue. I switched to babies and I thought, ‘I want babies’ and I think that might correlate with a little red blip but then I couldn’t sustain it … I’m wondering if I’m focusing so much that it’s just going blue because I’m focusing but I can’t get, I can’t get the self to kick in when I’m told to” (Figure 1B). In another run, the same meditator reported: “I tried to think about what was the thing that agitated me most and I thought it was [a certain person] and so I started thinking about her and I, at first it was just the name and I dropped into blue and so and then I started conjuring up images of [my boyfriend] with her and it super spiked [red] and then it just took a lot of effort so then I had to drop it. And I just kept trying to pick it up a little bit which I think correlates with the kind of like final two spikes, the kind of final two points in the red. Although, it was just so much energy, I couldn’t sustain it, which was why I couldn’t keep that really high spike going … I couldn’t sustain it and so that kind of correlates with not being able to hold on to that throughout” (Figure 1C).

As highlighted by these examples, a common theme that emerged from this study was that getting caught up in experience (e.g., “hold on”) rather than the content of experience itself increases PCC activity whereas present-centered awareness of mental content decreases PCC activity. Taken together, attention tasks when externally focused, studies of various types of meditation, and even a mindful stance toward an object (Taylor et al., 2011) suggest more precisely that PCC activity decreases when one becomes less caught up in ones experience, providing complementary evidence to studies showing its increase with tasks that elicit the opposite.

## Summary

The PCC seems to be involved in a number of modes of experience – for example, it is activated with evident experiences of getting caught up such as craving, and more subtle experiences of getting caught up, such as identifying with or being attached to attributes of ourselves. This hypothesized role for the PCC is also supported by data showing that the PCC decreases in activity when we are not caught up in experience, whether being focused on a task or meditating. Though we have brought together data from many realms of cognitive neuroscience to support this hypothesis, we by no means offer it as a definitive explanation, but instead an invitation for exploration and dialog; still no studies to our knowledge exist that directly test a role for the PCC in getting caught up in experience.

Given the growing evidence for the interconnected network nature of the brain, the PCC likely serves as a sentinel marker or as a node within a network of brain regions that together support or represent getting caught up in experience, for example, as a sub-component process of the DMN, rather than functioning in isolation. Such markers are helpful for then identifying and characterizing the networks that they represent. Studies using direct intracranial EEG recording have already begun to provide complementary neurophysiological data linking DMN activity to gamma frequency ranges (Jerbi et al., 2010; Dastjerdi et al., 2011; Ossandon et al., 2011; Foster et al., 2012). These and other modalities such as neurophenomenological methods are needed to directly assess how the being caught up in experience relates to PCC activity, to confirm and/or refine this and other plausible hypotheses that link PCC activity to cognitive processes and ultimately behavior.

## Conflict of Interest Statement

The authors declare that the research was conducted in the absence of any commercial or financial relationships that could be construed as a potential conflict of interest.
